# Tumor penetration and epidermal growth factor receptor saturation by panitumumab correlate with antitumor activity in a preclinical model of human cancer

**DOI:** 10.1186/1476-4598-11-47

**Published:** 2012-07-25

**Authors:** Daniel J Freeman, Kevin McDorman, Selam Ogbagabriel, Carl Kozlosky, Bing-Bing Yang, Sameer Doshi, Juan Jose Perez-Ruxio, William Fanslow, Charlie Starnes, Robert Radinsky

**Affiliations:** 1 Amgen Inc, Thousand Oaks, CA, USA; 2 Charles River Laboratories, Pathology Associates, Frederick, MD, USA

**Keywords:** EGFR, Monoclonal antibody, Xenografts, Pharmacokinetics, Pharmacodynamics

## Abstract

**Background:**

Successful treatment of solid tumors relies on the ability of drugs to penetrate into the tumor tissue.

**Methods:**

We examined the correlation of panitumumab (an anti-epidermal growth factor [EGFR] antibody) tumor penetration and EGFR saturation, a potential obstacle in large molecule drug delivery, using pharmacokinetics, pharmacodynamics, and tumor growth rate in an A431 epidermoid carcinoma xenograft model of human cancer. To determine receptor saturation, receptor occupancy, and levels of proliferation markers, immunohistochemical and flow cytometric methods were used. Pharmacokinetic data and modeling were used to calculate growth characteristics of panitumumab-treated tumors.

**Results:**

Treatment with panitumumab in vivo inhibited pEGFR, Ki67 and pMAPK levels vs control. Tumor penetration and receptor saturation were dose- and time-dependent, reaching 100% and 78%, respectively. Significant tumor inhibition and eradication (p < 0.05) were observed; plasma concentration associated with tumor eradication was estimated to be 0.2 μg/ml. The tumor inhibition model was able to describe the mean tumor growth and death rates.

**Conclusions:**

These data demonstrate that the antitumor activity of panitumumab correlates with its ability to penetrate into tumor tissue, occupy and inhibit activation of EGFR, and inhibit markers of proliferation and MAPK signaling.

## Introduction

Solid tumors differ from the normal tissue from which they were derived with respect to their vasculature, interstitial fluid pressure, lymphatic drainage, cell density, and extracellular matrix components [1]. This complex physiologic barrier can be especially challenging for large molecule therapeutics, such as targeted monoclonal antibodies. The intrinsic properties of antibodies such as the size of the therapeutic and affinity for the target may further hinder penetration into the tumor tissue. These properties must be balanced with the affinities of its competing ligands and the pharmacokinetic properties that result in clinically feasible dosing schedules [2,3].

Understanding the relationship among pharmacokinetic, pharmacodynamic, and anti-tumor parameters is critical for the development of an oncology therapeutic. It allows for the proper selection of dose and schedule of the molecule and the potential development of a clinically applicable marker of target coverage. Clinically, these correlations have proven to be challenging with the early small molecule tyrosine kinase inhibitors (SM TKIs) because of the variability in plasma and tumor exposure in patients and lack of biochemical coverage markers [4,5]. Although targeted monoclonal antibody therapeutics in general have substantially longer circulating half-lives, greater affinity and selectivity, and limited off-target toxicity compared with SMTKIs, one obstacle is achieving adequate exposure in solid tumors [3].

The epidermal growth factor receptor (EGFR) is a tyrosine kinase (TK) transmembrane receptor that is constitutively expressed in tissues of epithelial origin and is overexpressed in a variety of solid tumors including colorectal carcinoma, non-small cell lung carcinoma, renal cell carcinoma, ovarian, head and neck, prostate, breast, and pancreatic carcinomas [6,7]. Activation of the EGFR by EGF-like ligands mediates the Ras/Raf/MAPK, STAT and PI3K/AKT signaling pathways, which results in phenotypic changes including increased cellular proliferation, adhesion, migration, angiogenesis, and survival [8-10]. Furthermore, elevated expression of EGFR and its ligands have been found to be associated with poor clinical prognosis in several tumor types of epithelial origin [6,11,12].

Panitumumab is a fully human monoclonal antibody that binds EGFR with high affinity (5x10^-11^ M), prevents ligand-induced activation of all EGF-like ligands and production of angiogenic factors, and arrests tumor cell proliferation [13,14]. In preclinical studies, panitumumab treatment resulted in inhibition of tumor growth and eradication of tumors in some animal models [13,15,16]. Because panitumumab is a monoclonal antibody, it may have greater specificity for the EGFR compared with SM TKIs, which can cross-react with other relevant kinases [17]. Further, because panitumumab is fully human, it may also result in fewer immunogenic reactions in patients compared with chimeric or humanized EGFR monoclonal antibodies [13,15]. In clinical studies, panitumumab has demonstrated antitumor activity and a tolerable safety profile in colorectal cancer as a monotherapy and in combination with standard of care chemotherapeutics [18-20]. Selection based on tumor *KRAS* status has further increased the benefit of the patients treated with panitumumab [18,19,21,22].

To date, the extent of tumor penetration by panitumumab and its correlation with pharmacodynamic and antitumor activity has not been reported. Here, we investigated the correlation of serum levels of panitumumab, receptor occupancy of the EGFR, and inhibition of EGFR signaling with inhibition of cellular proliferation with antitumor activity in mouse model of human cancer.

## Materials and methods

### Animal studies

Six- to 10-week-old female CD1 nude mice (Charles Rivers Laboratories, Raleigh, NC) were used in all studies. Mice were housed in sterilized cages, 5 mice per cage, and were supplied *ad libitum* with Harlan Teklad Sterilized rodent diet 8656 and reverse-osmosis water from the institutional water supply system. Room temperature was maintained between 68–72° F, and relative humidity was maintained between 34 and 73%. The institutional laboratory housing the cages provided a 12-hour light cycle and met all Association for Assessment and Accreditation of Laboratory Animal Care (AAALAC) specifications.

A431 epidermoid carcinoma cells (American Type Culture Collection, Manassas, VA) were cultured in 10% fetal bovine serum (FBS)/RPMI to 80% confluency and harvested prior to injection. Mice were injected subcutaneously with 0.2 ml of 1 × 10^7^ A431 cells suspended in non-serum containing RPMI media into the left flank. Nine days following injection, mice were treated intraperitoneally with either panitumumab (5, 20, 200, or 500 μg), PBS vehicle control, or control IgG_2_ (500 μg) twice weekly. Tumor volumes, calculated as length × width × height in mm^3^, and body weights were recorded at regular intervals. Results were expressed as the mean ± standard error (SE). The data were statistically analyzed with factorial ANOVA followed by Scheffe's post hoc analysis for repeated measurements (StatView v5.0.1, SAS Institute). Mice were euthanized with CO_2_ asphyxiation, and for histological analysis, some tumors were harvested, immersion fixed, and embedded in paraffin using standard techniques. All experiments were conducted in accordance with institutional guidelines and under an Institutional Animal Care and Use Committee (IACUC) protocol.

### Immunoprecipitation and phosphorylation of EGFR

To assess EGFR phosphorylation in vitro, A431 carcinoma cells (80% confluent) were incubated in 0.5% FBS for 16 hours prior to treatment. Cells were treated with a control IgG_2_ antibody (10 μg/mL) or panitumumab (0.5, 2, and 10 μg/mL) for 60 minutes, followed by a 15-minute incubation with or without EGF (100 ng/mL). Cells were then washed three times in cold PBS and scraped in RIPA Buffer (20 mM Tris–HCl pH 7.5, 1% Igepal, 1% sodium deoxycholate, 150 mM NaCl, 0.1% SDS, 1% Triton X-100, pH 7.6). To measure EGFR phosphorylation in vivo, CD1 nude mice bearing A431 xenograft tumors of approximately 300 mm^2^ received intraperitoneal injections of either 1 mg of panitumumab or IgG_2_ control at both 24 hours and 4 hours prior to receiving 100 μg of EGF intravenously for 30 minutes. Tumors were excised and washed three times in cold PBS, and cell extracts were prepared in RIPA lysis buffer. EGFR was immunoprecipitated using an anti-EGFR monoclonal antibody clone, EGFR.1 (Ab-3 Labvision, Fremont, CA), in 500 μg of total cell extract. Phosphorylation of immunoprecipitated EGFR protein was then determined by immunoblot with an antiphosphotyrosine (pTYR) antibody (4G10 + pY99; Cell Signaling Technology, Beverly, MA). Immunoprecipitated EGFR was detected by immunoblot using an anti-EGFR antibody (#2232, Cell Signaling Technology, Beverly, MA).

### Pharmacokinetics

Serum samples for measuring panitumumab concentration for intraperitoneal doses administered (20, 200, or 500 μg) were collected postdose on 1, 2, 3, 4, 7, and 14 days after the initial dose and analyzed using an electrochemiluminescence (ECL) assay. Panitumumab in serum samples was captured using a biotinylated anti-idiotypic antibody to panitumumab immobilized on streptavidin-coated magnetic beads. This antibody was generated as described previously [23]. Panitumumab was detected with a ruthenium-labeled panitumumab anti-idiotypic antibody. ECL counts, which were directly proportional to panitumumab concentration, were measured with an IGEN M8 Analyzer (IGEN International Inc., Gaithersburg, MD). The observed serum panitumumab concentrations were analyzed using a compartmental approach. Because panitumumab does not bind mouse EGFR, EGFR-mediated clearance in mice is limited, and consequently, an open two-compartment PK model with first-order absorption from the site of administration and first-order elimination from the central compartment was fit to the observed panitumumab serum concentrations [13].

### Tumor penetration

A431 tumor xenografts from animals receiving control IgG_2_ antibody (500 μg) or panitumumab at doses of 20, 200, or 500 μg twice weekly were collected on days 1 and 4, fixed in IHC Zinc fixative (BD Pharmingen, San Diego, CA), and embedded in paraffin using standard techniques. Unstained 5 μm-thick tissue sections were deparaffinized, hydrated, and incubated with 20 μg/mL of an anti-idiotype antibody that specifically detects panitumumab (Amgen Inc., Thousand Oaks, CA) in DAKO antibody Diluent (DAKO, Carpinteria, CA) for 30 minutes. Slides were then incubated and labeled with 1:250 (2.4 μg/ml) alkaline phosphatase (AP)-conjugated goat anti-mouse antibody (Jackson ImmunoResearch Laboratories, West Grove, PA). AP Blue Substrate (Vector Labs, Burlingame, CA) was used to visualize the anti-idiotype antibody in the tumor samples. The EGFR pharmDx™ diagnostic kit (DAKO, Carpinteria, CA) was used to concurrently detect EGFR. Slides were quenched with 3% hydrogen peroxide, incubated with mouse anti-EGFR, and labeled with horseradish peroxide-conjugated dextran polymer. The red chromagen AEC (3-amino-9-ethylcarbazole; Vector Labs, Burlingame, CA) was used to visualize EGFR staining. Membrane staining intensity was graded by visual qualitative estimation of the amount of blue chromagen staining for panitumumab in tumor tissue compared with the intensity of red chromagen staining for EGFR. Tumor penetration was defined as the time and extent to which panitumumab enters into the tumor tissue.

### Saturation

The saturation level of EGFR by panitumumab was determined by flow cytometry on A431 epidermoid carcinoma cells. A431 cells were incubated in vitro with increasing concentrations of unlabeled panitumumab and phycoerythrin (PE)-labeled panitumumab (Invitrogen, Carlsbad, CA). Panitumumab was labeled with R-phycoerythrin (PE) and used at the lowest concentration required to achieve cell-surface binding saturation (1 μg/mL or 6.8 nM). Mouse anti-human EGFR monoclonal antibody (clone Ab-3 at 1 μg/ml; Labvision, Fremont, CA) was labeled with anti-mouse IgG-Alexa 488 and used to measure total EGFR expression on tumor cells. This antibody does not share the same epitope as panitumumab. A standard binding saturation curve was generated for using A431 cells grown in vitro. A431 cell suspensions were incubated with control human IgG_2_ or unlabeled panitumumab at 0, 0.21, 0.63, 1.83, 5.64, or 17 nM to compete with PE-labeled panitumumab kept constant at 6.8 nM. Simultaneously, cells were incubated with Alexa 488-labeled mouse anti-human EGFR antibody (Ab-3) at 6.8 nM for 1 hour in binding media (2% FBS, 1% normal rabbit serum, 10% normal goat serum, 0.1% sodium azide in PBS). Cells were analyzed for binding of PE-labeled panitumumab and Alexa 488-labeled anti-EGFR antibody (Ab-3) by 2-color flow cytometry using FACSCalibur (Becton Dickinson, Franklin Lakes, NJ). The ratiometric measure of bound PE-labeled panitumumab to total EGFR expression was calculated and normalized to 100% based on the standard saturation curve results. The standard curve was used to determine panitumumab-bound EGFR saturation. A decrease in the level of bound PE-labeled panitumumab as compared to total EGFR expression served as an indicator of bound unlabeled panitumumab. The relationship between EGFR saturation and panitumumab concentration were fitted to a hyperbolic E_max_ model to determine K_d_ values.

For in vivo panitumumab EGFR saturation analyses, tumor samples were collected from mice bearing A431 tumor xenografts treated with 500 μg of either panitumumab or control IgG_2_ antibody twice a week on days 0, 3, and 7. Tumor cell suspensions were extracted from individual tumor xenograft samples and resuspended by mincing the tumor pieces in a digestion buffer (collagenase 200 U/ml, DNAse 1500 U/ml, hyaluronidase 300 U/ml, and dispase 1 U/ml) for 20 minutes at 37° C. The isolated tumor cells were incubated with Alexa 488-labeled mouse anti-human EGFR antibody (Ab-3) and PE-labeled panitumumab at 6.8 nM each. The level of total EGFR expression and bound panitumumab was determined by flow cytometry as described above for A431 cells grown in vitro. Individual A431 tumor samples from 3 mice for each time point were analyzed and the standard error of the mean was provided.

### Immunohistochemistry

For the intracellular proliferation and signaling markers MIB-1 (Ki67) and phospho-MAPK (pMAPK), respectively, 5-μm-thick tissue sections were deparaffinized and hydrated. Slides were pretreated with Antigen Retrieval Citra (BioGenex, San Ramon, CA), then blocked with CAS Block (Zymed Laboratories, Inc., South San Francisco, CA) for 10 minutes. For Ki67, tissue sections were incubated for 1 hour with rabbit polyclonal anti-Ki67 (Novo Castra Laboratories Ltd., Newcastle upon Tyne, UK) at a dilution of 1:2000 followed by detection using biotinylated goat anti-rabbit immunoglobulin (Vector Laboratories, Burlingame, CA). pMAPK-blocked sections were incubated with rabbit polyclonal anti-phospho-p44/42 MAPK (Thr202/Tyr204; Cell Signaling Technology, Beverly, MA) at a dilution of 1:50, followed by detection using HRP-conjugated goat anti-rabbit antibody (Jackson ImmunoResearch Laboratories, West Grove, PA) at a dilution of 1:500. Slides were quenched with 3% hydrogen peroxide and followed with Avidin-Biotin Complex (Vector Laboratories, Burlingame, CA). Reaction sites were visualized with DAB (DAKO Corp., Carpinteria, CA) and the slides were counterstained with hematoxylin.

### Modeling tumor growth in an A431 carcinoma xenograft model

Tumor growth data were modeled using a modified version of the model proposed by Simeoni [24]. In the absence of treatment, tumor cells were assumed to proliferate at a constant rate. In the presence of panitumumab, an E_max_ model assumes that the concentration at the tumor induces damage in some cells eventually leading to cell death. In this model, E_max_ is the maximum cell death rate induced by blocking EGFR and EC_50_ is the concentration at the tumor that elicits 50% of maximum cell death rate. In addition, the concentration for tumor eradication was estimated from the model as previously described [24].

## Results

### Panitumumab inhibits ligand-induced EGFR phosphorylation in vitro and in vivo

**Figure 1:**
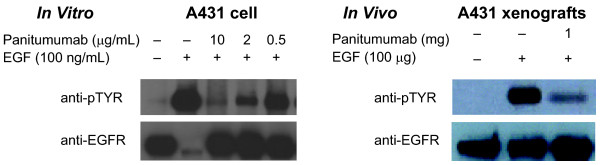
**Panitumumab inhibited ligand-induced pEGFR in vitro and in vivo.** Immunoprecipitation of both phospho- and total- EGFR harvested from (A) panitumumab-treated A431 epidermoid carcinoma cells in vitro 15 minutes after a treatment with EGF and (B) panitumumab-treated A431 xenograft tumors 30 minutes after an intraperitoneal injection of EGF.

To determine if panitumumab inhibits EGFR activation in A431 cells in vitro, serum-starved subconfluent cells were pretreated with panitumumab (or control IgG_2_) at varying concentrations and then stimulated with EGF for 15 minutes. Panitumumab treatment resulted in a dose-dependent inhibition of ligand-induced pEGFR (Figure 1A). Increasing concentrations of panitumumab resulted in a concomitant reduction in ligand-induced pEGFR at 10 μg/ml detected by immunoprecipitation and immunoblotting with anti-pTYR and anti-EGFR antibodies. EGF stimulation reduced total EGFR levels (Figure 1A).

To test if panitumumab can inhibit EGFR autophosphorylation in vivo, mice bearing A431 xenograft tumors of approximately 300 mm^3^ were injected intraperitoneally with 1 mg panitumumab or control IgG_2_ at 0 and 20 hours. Twenty-four hours post injection, mice were injected intravenously over 30 minutes with 100 μg EGF. Similar to the in vitro results, treatment with panitumumab resulted in an inhibition of ligand-induced pEGFR in A431 established tumor xenograft tissue as detected by immunoprecipitation and immunoblotting with anti-pTYR and anti-EGFR antibodies (Figure 1B).

### Pharmacokinetics of panitumumab in mice

**Figure 2:**
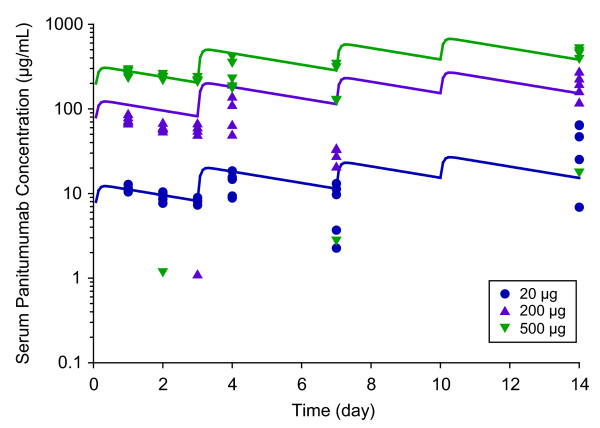
**The observed and modeled panitumumab PK profiles in xenograft mice after intraperitoneal administration of panitumumab twice a week.** Serum concentrations of panitumumab were assessed 1, 2, 3, 4, 7, and 14 days after the first dose (symbols, n = 5/time point); lines represent modeled pharmacokinetic profiles.

Panitumumab serum concentrations in the A431 xenograft-bearing mice after twice weekly intraperitoneal administration of panitumumab at 20, 200, and 500 μg were measured and fit well to the pharmacokinetic model (Figure 2). The maximum observed concentration (C_max_) and area under the curve (AUC) after the first dose based on the modeled curves increased in a dose-proportional manner. The C_max_ increased from 12.2 to 305 μg/mL and AUC increased from 30.2 to 755 μg·day/mL as the dose increased from 20 to 500 μg/kg. Absorption rate, central volume of distribution, and systemic clearance were estimated to be 0.54 h^-1^, 2.61 mL, and 3.11 mL/day, respectively.

### Panitumumab penetrates xenograft tissues in a dose- and time-dependent manner

**Figure 3:**
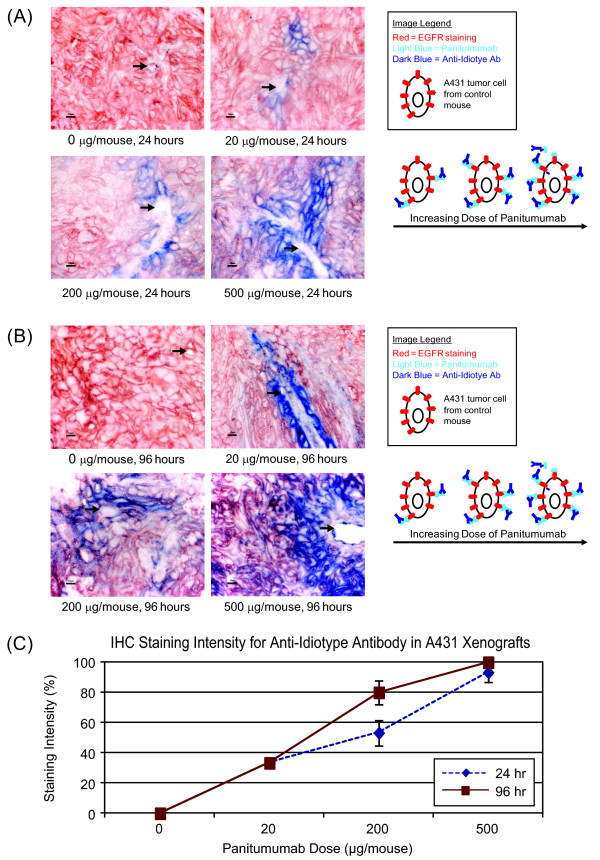
**Penetration of panitumumab into tumor xenograft tissue is dose and time dependent.** Panitumumab initially surrounded the afferent blood supply and then penetrated diffusely into the surrounding tumor tissue. A431 tumor xenograft samples of animals receiving 0, 20, 200, or 500 μg panitumumab were collected **(A)** 24 hours or **(B)** 96 hours after initiation of dosing and incubated with anti-idiotype IgG_2_-bound panitumumab to detect administered panitumumab (blue chromagen) and DAKO anti-EGFR to detect EGFR (red chromagen). Micron bars = 10 μm all high magnification images and black arrows identify blood vessels. **(C)** IHC staining intensities for the anti-idiotype IgG_2_-bound panitumumab were qualitatively graded and plotted as an estimated percent of total staining intensity.

The ability of panitumumab to penetrate tumors was investigated in mice bearing A431 xenografts. Animals bearing established tumors of approximately 300 mm^3^ were treated with panitumumab at 20, 200, or 500 μg via intraperitoneal injection. Tumors were harvested and analyzed for the degree of panitumumab penetration at 24 or 96 hours post injection. Staining for panitumumab was initially more intense around blood vessels and in the peripheral regions of the tumor tissue where blood flow is the highest. Panitumumab staining increased into the surrounding tissues with increased dose and time. At 24 hours, staining for panitumumab was observed and the intensity/extent was dose-dependent: ~37% with 20 μg, ~53% with 200 μg, and ~93% with 500 μg (Figure 3A). At 96 hours, staining became more diffuse with ~37% staining at 20 μg, ~80% at 200 μg and ~95% at 500 μg (Figure 3B). Using qualitative immunoreactivity grading, maximum tumor penetration of greater than 95% was reached with 500 μg of panitumumab after 96 hours (Figure 3C).

### Panitumumab saturates EGFR on A431 epidermoid carcinoma cells in vitro and in vivo

**Figure 4:**
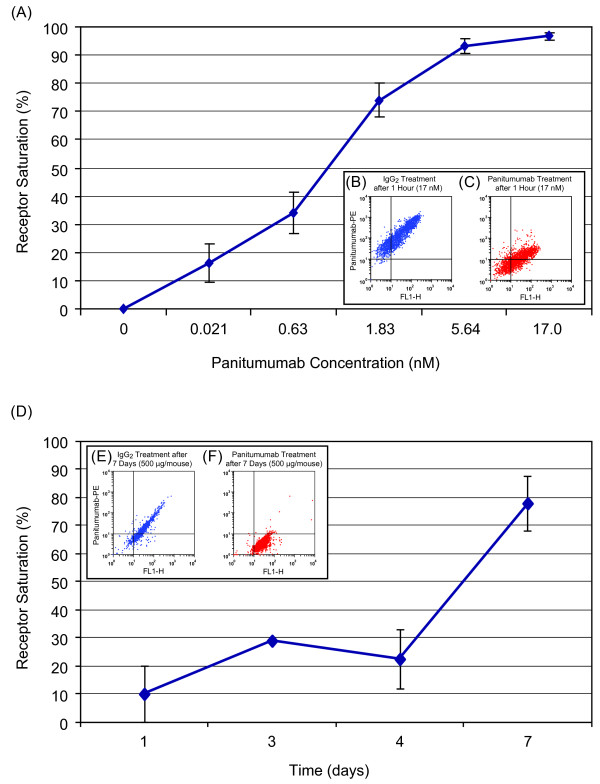
**Treatment with panitumumab resulted in the saturation of EGFR on A431 epidermoid carcinoma cells in vitro and in vivo in xenograft tumors as determined by flow cytometry. (A)** A431 cells were incubated in vitro with increasing concentrations of unlabeled panitumumab and phycoerythrin (PE)-labeled panitumumab to determine lowest concentration required to achieve cell-surface binding saturation. **(B, C** [inset]) FACS dot plots of PE-panitumumab vs Alexa-EGFR of A431 cells treated with 17 nM of either *B*, control IgG or *C,* panitumumab for 1 hour. **(D)** The percent of EGFR saturation following treatment was determined in vivo by measuring the level of bound panitumumab on the dissociated tumor xenograft cells by flow cytometry and plotting test results against the standard curve generated in Figure 4A. **(E, F** [inset]) FACS dot plots of PE-panitumumab vs Alexa-EGFR of dissociated A431 xenograft cells treated with 500 μg of either *E*, control IgG or *F,* panitumumab; tumor cells were dissociated and assayed 7-days post-treatment.

To determine the EGFR saturation (receptor occupancy) in A431 cells following treatment with panitumumab in vitro and in vivo, a flow cytometry assay was developed using a non-competing Alexa 488-labeled mouse anti-human EGFR antibody and PE-labeled panitumumab. The ratio of Alexa 488-labeled antibody (which measures the total amount of EGFR on A431 cells) compared with PE-labeled panitumumab (which competes for unlabeled panitumumab) allowed for the determination of the level of panitumumab-bound EGFR and hence saturation. The saturation curve showed that a panitumumab concentration of 6.8 nM was sufficient to saturate greater than 90% of expressed EGFR on A431 cells in vitro whereas 17 nM was sufficient to saturate 97% (range 95.6 to 98%; Figure 4A). FACS dot plots of PE-panitumumab vs Alexa-EGFR of A431 cells treated with control IgG (Figure 4B) or unlabeled panitumumab (Figure 4C) demonstrated the binding specificity of panitumumab to EGFR.

Using the in vitro standard curve, the EGFR saturation concentration in vivo was assessed in dissociated cells from A431 xenografts from mice treated with 500 μg panitumumab or control IgG_2_ antibody twice weekly (Figure 4D). Saturation was assessed on days 1, 3, 4, and 7 after treatment. Administration of panitumumab at 500 μg resulted in the saturation of EGFR expressed in A431 xenografts in a time-dependent manner, with a mean saturation of 10% at day 1, 30% at day 3, 22.5% at day 4, and 78% at day 7 (Figure 4D). The estimated (SE) Kd value was 0.922 (0.059). Similarly, FACS dot plots of PE-panitumumab vs Alexa-EGFR of A431 cells treated with control IgG after 7 days (Figure 4E) or panitumumab after 7 days (Figure 4F) demonstrated the binding specificity of panitumumab to EGFR in the assay.

### Panitumumab reduces markers of proliferation in established A431 xenografts

**Figure 5:**
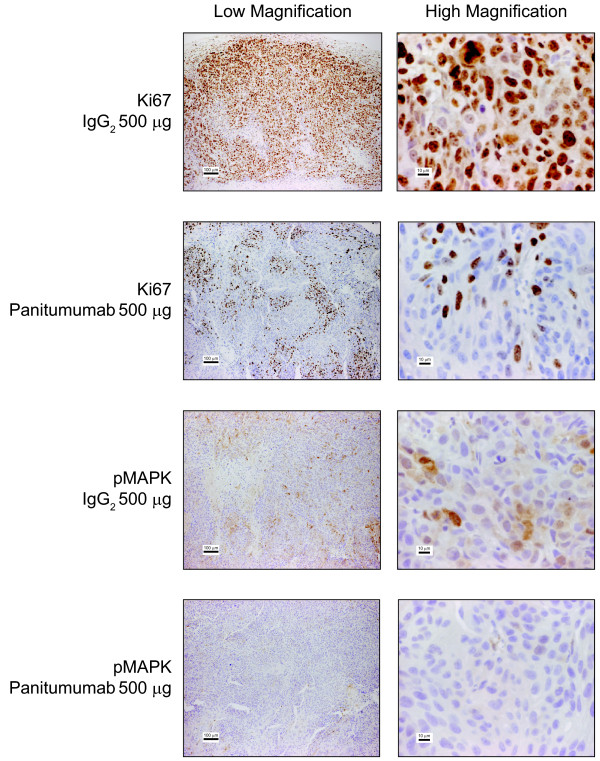
**Markers of proliferation (Ki67) and downstream kinase signaling (pMAPK) in A431 epidermoid carcinoma xenografts were decreased after treatment with panitumumab.** Fixed tissue sections were prepared from A431 xenograft tumors from mice treated with either 500 μg IgG_2_ control vehicle or 500 μg panitumumab twice a week for two weeks. Qualitative changes in Ki67 or pMAPK immunoreactivity were visualized with DAB and hematoxylin counterstain. Micron bars = 100 μm for low magnification images on the left and 10 μm for high magnification images on the right. Representative fields are shown.

Ligand-induced activation of the EGFR can induce cellular proliferation via the MAPK signaling pathway. To determine if panitumumab can inhibit cellular proliferation in vivo, mice bearing established A431 tumor xenografts were treated twice a week for 14 days with 500 μg of either panitumumab or IgG control. Fixed tissue sections were evaluated for levels of cellular proliferation and signaling markers, Ki67 and pMAPK. Panitumumab treatment of A431 xenograft tumors resulted in a reduction in Ki67 and pMAPK staining compared with the vehicle control (Figure 5). These data suggest that panitumumab mediates inhibition of EGFR activity by decreasing cellular proliferation and downstream MAPK signaling.

### Panitumumab inhibits growth of established A431 xenografts in a dose-dependent manner

**Figure 6:**
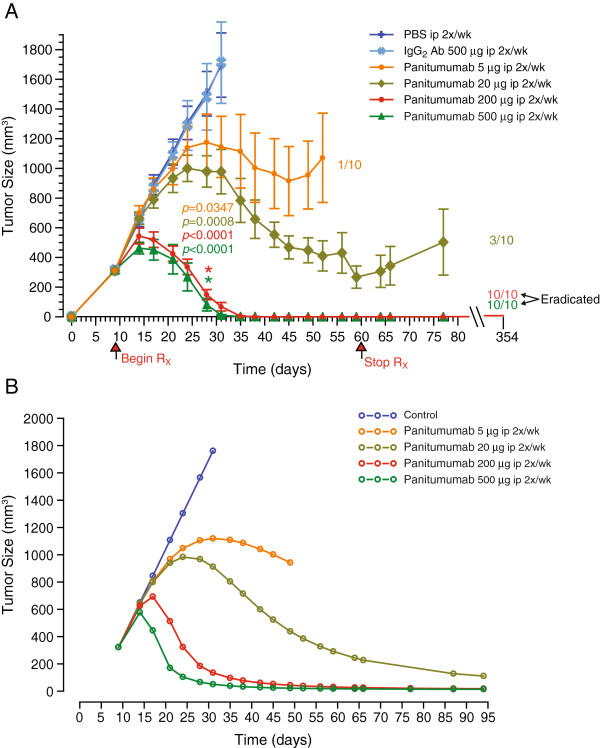
**Observed and model-fitted tumor growth curves in an A431 carcinoma xenograft model. (A)** Mice with established A431 tumors received PBS (purple line), human IgG_2_ 500 μg (blue line), panitumumab 5 μg (orange line), 20 μg (brown line), 200 μg (red line) or 500 μg (green line) for 52 days. Mice were monitored continuously for 300 days after the last dose of panitumumab was administered. **(B)** Tumor inhibition modeled data.

To determine if tumor penetration, EGFR saturation, and inhibition of EGFR activation and proliferation correlated with anti-tumor activity, mice bearing A431 xenograft tumors of approximately 300 mm^3^ tumors were injected intraperitoneally twice a week for 50 days with PBS, 500 μg of control IgG_2_ antibody, or 5, 20, 200 or 500 μg of panitumumab (n = 10 animals in each treatment group). Treatment with panitumumab resulted in a dose-dependent tumor inhibition at the 5- and 20-μg doses and in complete tumor eradication at the 200- and 500-μg doses. Control animals were euthanized on day 22 whereas animals treated with panitumumab at 5 μg and 20 μg were euthanized on days 44 and 67, respectively, because of uncontrolled tumor growth and consistent with IACUC guidelines. In animals treated with panitumumab at 200 μg and 500 μg, no tumors were detected by day 28 of treatment. These mice remained disease free for an additional 300 days after the last dose was administered (Figure 6A), at which time they were euthanized and no further data were collected. No difference in the body weights between the control-treated and panitumumab-treated animals were observed (data not shown).

The observed tumor growth data from the A431 xenograft study (Figure 6A) were modeled to calculate the growth and death rates upon treatment with panitumumab. This model described a mean A431 tumor cell growth of 3.73 mL/h, which was consistent with the observed results. Maximum EGFR-mediated tumor cell death rate was 8.97 h^-1^ and the steady-state concentration at the tumor that elicits 50% of maximum cell death rate was 0.81 μg/mL (Figure 6B). In addition, the concentration for tumor eradication, which accounts for both tumor growth and tumor death was estimated to be 0.20 μg/mL.

## Discussion

The data presented here examined the correlation of panitumumab tumor penetration and EGFR saturation, a potential obstacle in drug delivery of large molecules in treating solid tumors, using pharmacokinetics, pharmacodynamics, and anti-tumor activity in an A431 epidermoid carcinoma xenograft model system.

One important factor that leads to the clinical efficacy of a therapeutic is its ability to modulate the target for which it is intended. Although A431 cells express approximately 1.2 million EGFRs per cell [13], there is only a minimal amount of basal phosphorylation of the EGFR in vitro or in vivo (Figure 1). Therefore, to address panitumumab target coverage, we employed an inhibition of ligand-induced phosphorylation assay. Panitumumab treatment inhibited EGFR autophosphorylation in A431 cells in vitro in a dose-dependent manner as well as in vivo in the A431 xenograft model. It has been shown that activation of EGFR by EGF resulted in rapid internalization and degradation of the receptor [25,26]. Our data demonstrated similar reductions in the total EGFR levels upon EGF stimulation (Figure 1A). In vivo, two treatments with panitumumab were sufficient to significantly inhibit EGFR autophosphorylation in the A431 cells growing as xenografts. Although detectable levels of phosphorylated EGFR remained in the tumors, this may be explained by an incomplete penetration of the antibody at the 24-hour time point (see Figure 3A). The significant inhibition of EGFR phosphorylation may also suggest that EGF penetration is restricted to the perivascular space at this early time point.

Panitumumab serum concentrations from tumor-bearing mice increased in a dose-proportional manner. The trough levels in the xenograft bearing mice at a dose of 200 μg were similar to those observed in the clinical setting [21]. Using concurrent pharmacodynamic and pharmacokinetic data, panitumumab penetration and EGFR saturation in the tumors was measured. Barriers to tumor penetration include high interstitial fluid pressure and hydrostatic vascular pressure. Although these potential pressures were not measured in this study, panitumumab was able to penetrate the tumors. However, with the lower doses and at early time points, penetration of panitumumab was restricted to regions of tumor tissue adjacent to the afferent blood supply which has been observed with other antibodies [2,27].

Consistent with the dose-dependent increase in serum levels, panitumumab penetrated tumors in a dose- and time-dependent manner. After 24 hours with the lowest dose of 20 μg, panitumumab was detected in tumor samples. Increasing panitumumab doses resulted in increased tumor penetration to approximately 95% with the highest dose of 500 μg 96 hours after the initial injection. Although significant levels of panitumumab bound to the EGFR on the tumor cell surface were measured at the single cell level at day 7 post treatment in the receptor saturation assay (Figure 4B), there was some variation between the results obtained with the tumor penetration assay and the receptor saturation assay at the earlier time points, which may reflect how the total panitumumab was detected in each assay. The tumor saturation assay only measures the level of panitumumab on the cell surface and would not account for receptor internalization as a result of panitumumab treatment [28]. In addition, although significant effort was made to minimize processing time, at the earlier time points when panitumumab concentrations are lower, the multiple processing steps for the tumor saturation assay may wash off panitumumab versus immediate and direct fixation for the tumor penetration assay.

Panitumumab administration resulted in dose-dependent tumor regression and eradication in this A431 xenograft model, with animals remaining free of disease for 300 days off treatment. Interestingly, 100% tumor eradication was seen at a dose of 200 μg twice a week. The serum exposure of panitumumab associated with these animals was similar to those achieved in patients [21]. This association between drug exposure that is achievable in the clinic and response in preclinical models is different than that seen for some of the small molecule EGFR inhibitors, which might explain the lack of activity in settings that express only wild-type EGFR [29,30].

## Conclusions

These preclinical studies indicate that the pharmacokinetic and pharmacodynamic parameters of panitumumab correlated with in vivo antitumor activity. Furthermore, understanding these parameters may help to understand the responses seen in patients receiving panitumumab treatment.

## Competing interests

DJF, SO, B-BY, SD, JJP-R, WF, CS, and RR are employees of and shareholders in Amgen Inc. KM and CK were employees of and shareholders in Amgen Inc. at the time this research was conducted. This study was funded by Amgen Inc.

## Authors’ Contributions

DJF conceived of the study, and participated in its design and coordination, and helped draft the manuscript. KM performed the pathology analysis and interpretation and helped draft the manuscript. SO performed the molecular analysis. CK performed the in vivo pharmacodynamic analysis. B-BY performed the in vivo pharmacokinetic analysis. SD performed the pharmacokinetic modeling. JJP-R carried out the pharmacokinetic/pharmacodynamic/efficacy modeling. WF carried out the tumor saturation analysis and interpretation. CS developed the in vivo efficacy model. RR participated in design and coordination of the study and helped draft the manuscript. All authors read and approved the final manuscript.
